# Assessment of Awareness and Knowledge of Proton Pump Inhibitors Among the General Population in the Qassim Region, Saudi Arabia

**DOI:** 10.7759/cureus.46749

**Published:** 2023-10-09

**Authors:** Reema Almuzaini, Ahmed S Almuzaini, Abdullah Mohammed Alqifari, Asma Alsohaibani, Latifah Y Almutlaq, Raghad Alwehaibi, Razan S Alfurayji, Thekra A Alsamel

**Affiliations:** 1 College of Medicine, Qassim University, Buraydah, SAU; 2 Collage of Medicine, Qassim University, Buraydah, SAU; 3 Gastroenterology, King Fahad Specialist Hospital, Buraydah, SAU; 4 College of Medicine, Qassim University, Qassim, SAU; 5 Obstetrics and Gynaecology, Qassim University, Buraydah, SAU

**Keywords:** widespread use of ppis, over-utilization of ppis, optimal timing of ppis, saudi arabia, side effects of ppis, proton pump inhibitors

## Abstract

Objective: This descriptive observational cross-sectional study aimed to assess the general population's awareness, attitudes, and behaviours towards proton pump inhibitor (PPI) usage, as well as their knowledge about associated side effects, in the Qassim region of Saudi Arabia.

Methods: An autonomous online survey was conducted from June 15, 2023, to September 1, 2023, using social media. The survey targeted adult residents of Qassim and collected a total of 1090 respondents. Data analysis employed descriptive statistics, chi-square tests, and probit regression using R version 4.3.1 (RStudio, Boston, MA). A significance level of p<0.05 was utilized to interpret the results.

Results: A total of 1050 samples, limited to residents of Qassim, were analyzed. Significant associations were observed between awareness of PPIs and factors such as side effects (adjOR = 1.19, 99% CI: 1.08-1.31), widespread PPI use (adjOR = 1.24, 99% CI: 1.12-1.38), PPI usage (adjOR = 2.47, 99% CI: 2.18-2.82), and optimal PPI timing (adjOR = 1.30, 99% CI: 1.13-1.50). Additionally, age, educational attainment, and employment in the medical field significantly influenced awareness gaps related to potential side effects, PPI prevalence, adherence to medical prescriptions, and optimal timing for PPI usage.

Conclusion: The current study concludes that a significant portion of individuals in the Qassim region lack awareness regarding the potential side effects of PPI usage. Comprehensive healthcare education is required to bridge awareness gaps regarding PPIs and foster informed medication practices. By grasping the intricacies of individual perceptions, medical engagement, and demographic factors, healthcare providers and policymakers can collaboratively empower individuals in the Qassim region to make informed choices regarding their health and medication usage.

## Introduction

Proton pump inhibitors (PPIs), introduced in 1989 with the discovery of omeprazole and now globally prevalent due to their proven efficacy and tolerability, are a common prescription choice. However, their uptake is influenced by attitudinal factors, where positive perceptions encourage usage, while concerns about side effects, long-term use, or dependency can lead to hesitancy [1]. Physicians, including geriatricians, significantly affect PPI prescription rates, with studies revealing correlations between their prescription behaviour, hospital grade, education level, and participation in educational programmes, highlighting clinical limitations [2,3]. Negative attitudes contribute to PPI overuse, as healthcare providers in one study believed it was widespread due to insufficient understanding of side effects among doctors and patients [4]. Behavioural factors, including effective communication and higher health literacy, positively influence PPI adherence, while nurses' attitudes towards PPI usage vary, with regional differences [4]. Compliance with prescribed PPI regimens is crucial; one study found incorrect administration, emphasizing the need for evidence-based interventions. Perceived needs influence usage, driven by symptom severity or medical history [5].

Literature review

The number of people relying on PPIs in the United Kingdom has risen significantly, from 32.6 million in 2008 to 60 million in 2018, according to data from [6]. The over-utilization of these medications is regarded as a substantial financial burden for both the government and the general community, resulting in an estimated cost of around £2 billion per year on a global scale, as indicated by research conducted by Aljahdli’s [7].

PPIs bind permanently to the gastric hydrogen-potassium ATPase pump, commonly called the proton pump in stomach parietal cells, deactivating it and inhibiting gastric acid secretion, a mechanism explained by Aljahdli [7]. They are extensively used to treat and prevent upper gastrointestinal tract conditions, including peptic ulcers, GERD, eradicating Helicobacter pylori, managing dyspepsia, countering NSAID-induced ulcers, and addressing Barrett's oesophagus, as discussed by Ahmed and Clarke [8].

In their comprehensive study on GERD pathophysiology, diagnosis, and treatment, Chen and Brady [9] emphasized the need for most PPIs to be taken 30 minutes before meals, except for omeprazole, which can be taken before or after meals. The clinical investigation by Yoon et al. [10] regarding the impact of tegoprazan 50 mg (a potassium-competitive acid blocker) on healthy males found that meal timing had no clinically significant effects on its pharmacokinetics, efficacy, or safety, concluding it can be administered without meal considerations. Additionally, the study by Laine et al. [11] in the USA on H. pylori-negative subjects evaluating a potassium-competitive acid blocker (vonoprazan) and a PPI (lansoprazole) reported that PPIs are more effective when taken shortly before meals.

The evidence suggests frequent overuse of PPIs, with 25-70% of prescriptions lacking proper indications, often due to their accessibility as over-the-counter drugs, as explored by Jaynes and Kumar [12]. Excessive PPI use surpasses documented gastrointestinal symptom cases. While PPIs effectively treat acid-related conditions, their excessive off-label use leads to severe adverse events [13]. Expert analysis by Savarino et al. [14] highlights ongoing inappropriate PPI use in Europe, driven by improper hospital prescriptions and post-discharge continuation.

In a Spain-based study by Savarino, over 50% of PPI prescriptions were deemed inappropriate, spanning hospitals and primary care settings, as revealed in their examination of PPIs' suitable application [15]. In a comprehensive systematic review, Scarpignato et al. [16] noted that despite overuse and inappropriate employment of PPIs challenging their safety profile, adverse events typically occur at a frequency of 1-3%, including headaches, nausea, abdominal pain, constipation, flatulence, diarrhoea, rash, and dizziness.

Prolonged PPI use alters gastric pH, fostering microorganism growth and increasing gastrointestinal infection risk. PPI use is linked to osteoporosis and fractures. The Beers criteria recommend limiting PPIs in elderly patients to eight weeks, supported by Freedberg's research [17]. Analyzing UK Health Improvement Network (THIN) data, Freedberg et al. [18] found higher PPI exposure in young adults compared to children, indicating a greater fracture risk among young adults.

Despite PPIs being generally well tolerated, a range of studies have highlighted their link to various adverse effects. According to certain investigations, patient awareness concerning the adverse effects linked to PPIs remains limited, indicating that a significant majority of participants were oblivious to any potential adverse effects of these medications, as indicated in the works of Hamurtekin et al. [19] and White et al. [20].

Socio-demographic status significantly influences sustained PPI use, as shown in the Netherlands study by Van Boxel et al. [21]. Low educational attainment correlates with chronic PPI utilization, emphasizing education's role in health-related attitudes and knowledge [21]. Similarly, a nationwide Danish cohort study by Haastrup et al. [21] linked long-term PPI use to lower income and education levels.

Despite the acknowledged effectiveness of PPIs, these scenarios have collectively contributed to the widespread occurrence of improper PPI utilization. A study conducted within an academic hospital in Saudi Arabia documented a substantial prescription rate of 57.6% for PPIs, as reported by Basheikh [22].

In a comprehensive medication usage study conducted by AlKhamees et al. [23] on the top ten most commonly used drugs in Saudi Arabia from 2010 to 2015, antibiotic and analgesic use predominated, followed by PPIs and anti-diabetic medications. The consumption of anti-hyperlipidemic drugs and treatments for erectile dysfunction followed. The study also noted an increasing trend in the use of pantoprazole and esomeprazole in Saudi Arabia during this period. While pantoprazole had higher overall usage than esomeprazole, there was a period between 2012 and 2013 when pantoprazole surpassed esomeprazole in utilization.

The study by Asdaq et al. [24] in Riyadh, Saudi Arabia, involving medical doctors, pharmacists, and nurses from diverse public and private hospitals, found that individuals with higher education, middle-aged individuals, and those with more professional experience had greater knowledge levels. Pharmacists and nurses showed lower reliance on PPIs compared to doctors. The research also revealed a positive correlation between healthcare professionals' attitudes, knowledge, and behaviour regarding PPI usage in Saudi Arabia.

Another study by Alasmari et al. [25] focused on physicians, pharmacists, and clinical pharmacists in Saudi Arabia, revealing significant disparities in PPI awareness, with more reporting poor knowledge. AlShammari et al. [26] studied adults in Saudi Arabian malls, assessing over-the-counter PPI usage. Initially, low knowledge and awareness improved through pharmaceutical advertising, enhancing public understanding of gastroesophageal reflux disease. (GERD) Alzahrani and Al Turki [27] studied Riyadh residents trained in family and internal medicine. Findings showed insufficient GERD management knowledge and age-linked better understanding. Most residents are prescribed PPIs before meals, but for a shorter duration.

Therefore, the purpose of this study is to assess the awareness, attitude, and behaviour regarding PPI usage among the general population of the Qassim region in Saudi Arabia and to assess if there is long-term improper use of PPIs in the population and their knowledge about its side effects through a cross-sectional study design using a validated self-administered questionnaire.

## Materials and methods

The present investigation employed a descriptive observational cross-sectional study design. An autonomous online survey was undertaken amongst adult residents of Qassim (age ≥ 18). This survey was administered via diverse social media platforms from June 15, 2023, to September 1, 2023. All readily accessible populations conforming to the eligibility criteria were extended invitations to participate in the inquiry.

A targeted minimum sample size of 385 was determined through the utilization of the following formula: n = (z)² p (1 − p)/d², wherein the sample size, denoted as 'n', p = 50%, and the confidence level is set at 95%, thus yielding a Z score of 1.96. The margin of error (E) is established at 5%, and the population size stands at 100,000. The non-probability sampling method, namely convenience sampling, was implemented. The study participants were encompassed based on their convenient accessibility and willingness to partake in our research endeavour.

The study encompassed individuals who, having reached the age of 18 years or more, were inhabitants of the Qassim region. The recruitment process focused on those who willingly signed the informed consent document. An ethical approval letter was sought from the Qassim University IRB with the number 44-018699.

A validated questionnaire, translated into Arabic, was employed to gauge the populace's awareness, attitude, and conduct concerning the usage of PPIs and their understanding of associated side effects. Various sections of the questionnaire encompassed demographic details, knowledge, and attitudes towards PPIs (their usage and adverse effects). Table 1 presents the survey questions and their corresponding categories.

The data underwent meticulous cleansing and scrutiny to rectify any potential errors or discrepancies. The analysis of the data was carried out using the R programming language, specifically version 4.3.1 (RStudio, Boston, MA) [28]. The relevant descriptive statistics were computed and then summarized in terms of frequency, percentage, and mean values. The analysis of categorical variables, stratified by demographics, was conducted using the Chi-square test. Odds ratios, both unadjusted and adjusted, were calculated via probit regression to assess the risk factors. Statistical significance was indicated for differences where the p-value was less than 0.05.

**Table 1:** The questionnaire

Question	Explanation	Construct
Have you ever heard of antacids/PPIs? (Nexium - Esmoperazole - Pantoparazole - Omeperazole - Omez Gasec)	It asks about familiarity with antacids/PPIs, showing awareness or knowledge.	Awareness
Have you ever used one of these medications?	It asks about personal experience with these medications.	Usage
In case you used the medication, what was the indication for using the drug?	Falls under knowledge. It seeks reasons for using the medication.	Knowledge
Duration of using the medication?	It falls under behaviour. It inquires about the length of medication use.	Behaviour
In case you used the medication, did you complete the course?	Pertains to behaviour. It asks if the prescribed course was finished.	Behaviour
If you answered No to Q5, Why?	Addresses attitude. It seeks reasons behind not completing the course.	Attitude
Based on your knowledge, which of the following is a possible side effect of PPI?	Is about knowledge. It tests understanding of side effects.	Knowledge
In your opinion, what time is most suitable for taking the medication?	Is about usage. It addresses practical usage.	Usage
In your opinion, do you think PPIs are widely used in Saudi Arabia?	Is about knowledge. It asks about prevalence.	Knowledge
Do you think short-term use of PPI couldn't cause side effects?	Is about attitude. It seeks your opinion on short-term use effects.	Attitude

## Results

The subsequent section presents the findings in terms of descriptive (Table 2) and multivariate analyses (Tables 3-7), along with corresponding figures. The primary analytical approach entails utilizing the probit regression model, wherein all the statistically significant variables indicated by chi-statistics are incorporated as independent variables, while awareness of antacids/PPIs is considered the dependent variable.

**Table 2:** Socio-demographic characteristics and awareness of antacids/PPIs (Nexium, Esmoperazole, Pantoparazole, Omeprazole, Omez, Gasec) (n=1090) Notes: The data are presented in the form of frequencies (n) and percentages (%). PPI: proton pump inhibitor.

Socio-demographic	Characteristic	No	Yes	p-value
Residence Qassim Region	No	11 (1%)	21 (1.9%)	0.374
	Yes	447 (41%)	611 (56.1%)	
Nationality	Non-Saudi	9 (0.8%)	27 (2.5%)	0.035
	Saudi	449 (41.2%)	605 (55.5%)	
Gender	Female	245 (22.5%)	333 (30.6%)	0.793
	Male	213 (19.5%)	299 (27.4%)	
Age	<20 years	79 (7.2%)	54 (5%)	0.001
	21-30 years	184 (16.9%)	253 (23.2%)	
	31-40 years	74 (6.8%)	123 (11.3%)	
	41-50 years	87 (8%)	148 (13.6%)	
	51-60 years	29 (2.7%)	47 (4.3%)	
	>61 years	5 (0.5%)	7 (0.6%)	
Marital status	Single	239 (21.9%)	302 (27.7%)	0.134
	Married	209 (19.2%)	301 (27.6%)	
	Divorced	7 (0.6%)	21 (1.9%)	
	Widow/Widower	3 (0.3%)	8 (0.7%)	
Education level	Less than high school	42 (3.9%)	31 (2.8%)	0.015
	High school	104 (9.5%)	160 (14.7%)	
	Diploma	42 (3.9%)	57 (5.2%)	
	Bachelor	258 (23.7%)	351 (32.2%)	
	Higher degree	12 (1.1%)	33 (33%)	
Occupation	Unemployed	85 (7.8%)	107 (9.8%)	0.196
	Student	163 (15%)	196 (18%)	
	Employee	190 (17.4%)	291 (26.7%)	
	Retired	20 (1.8%)	38 (3.5%)	
Worked in the medical field	No	413 (37.9%)	417 (38.3%)	<0.001
	Yes	45 (4.1%)	215 (19.7%)	
Used PPIs medication	I have never used it	432 (39.6%)	267 (24.5%)	<0.001
	Yes, without medical consultation	5 (0.5%)	103 (9.4%)	
	Yes, with medical consultation	21 (1.9%)	262 (24%)	
Suitable time for taking PPIs	After meal	261 (23.9%)	189 (17.3%)	<0.001
	Before meal	152 (13.9%)	404 (37.1%)	
	With meal	45 (4.1%)	39 (3.6%)	
PPIs are widely used in KSA	I do not know	184 (16.9%)	101 (9.3%)	<0.001
	No	50 (4.6%)	39 (3.6%)	
	Yes	224 (20.6%)	492 (45.1%)	
Short-term use and side effects	I do not know	192 (17.6%)	162 (14.9%)	<0.001
	No	67 (6.1%)	96 (8.8%)	
	Yes	199 (18.3%)	374 (34.3%)	

Descriptive statistics

Table 2 presents a comprehensive overview of socio-demographics categorized by awareness of antacids/PPIs. Notably, the demographics were not classified based on specific antacids/PPIs such as Nexium, Esomeprazole, Pantoprazole, Omeprazole, Omez, or Gasec. The study included a total of 1090 participants in the sample. It is important to note that the study specifically focused on residents of the Qassim region. After data cleaning, 32 participants who were non-residents of Qassim were removed, resulting in a final sample size of 1058 participants. This final sample size was used for conducting the probit analysis.

**Figure 1:**
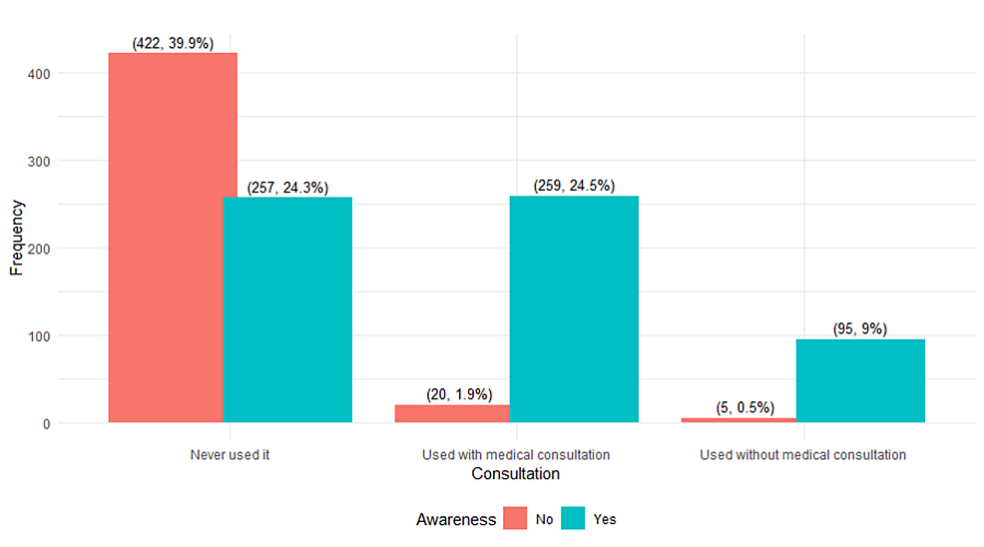
Comparing awareness of PPIs and their usage Notes: The data are presented in the form of frequencies (n) and percentages (%). PPI: proton pump inhibitor.

Figure 1 provides a comparative visualization of the awareness of PPIs and their usage. The comparison is established among individuals who have never used them, those who have used them with medical consultation, and those who have used them without medical consultation.

**Figure 2:**
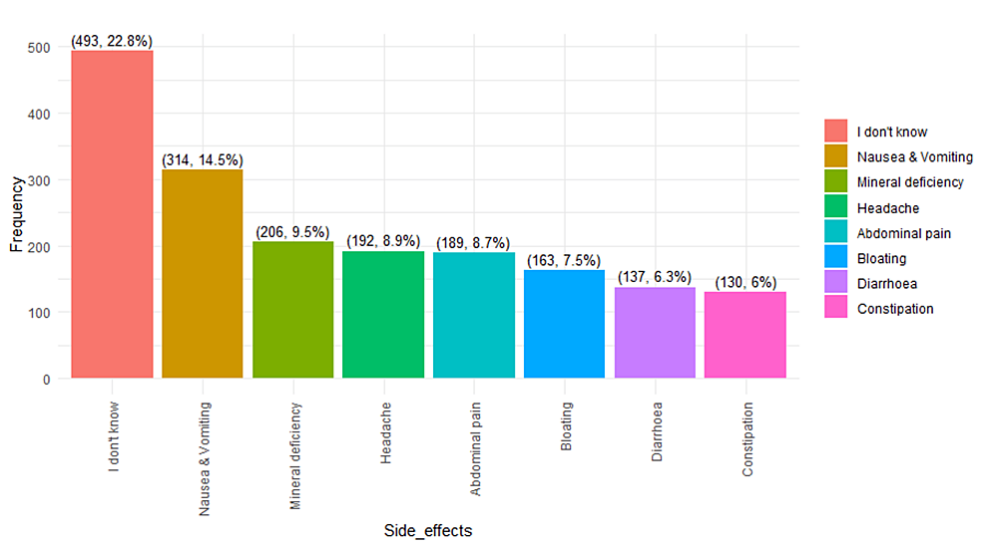
Top 8 side effects of PPI usage as identified in the study Notes: The data are presented in the form of frequencies (n) and percentages (%). PPI: proton pump inhibitor.

**Figure 3:**
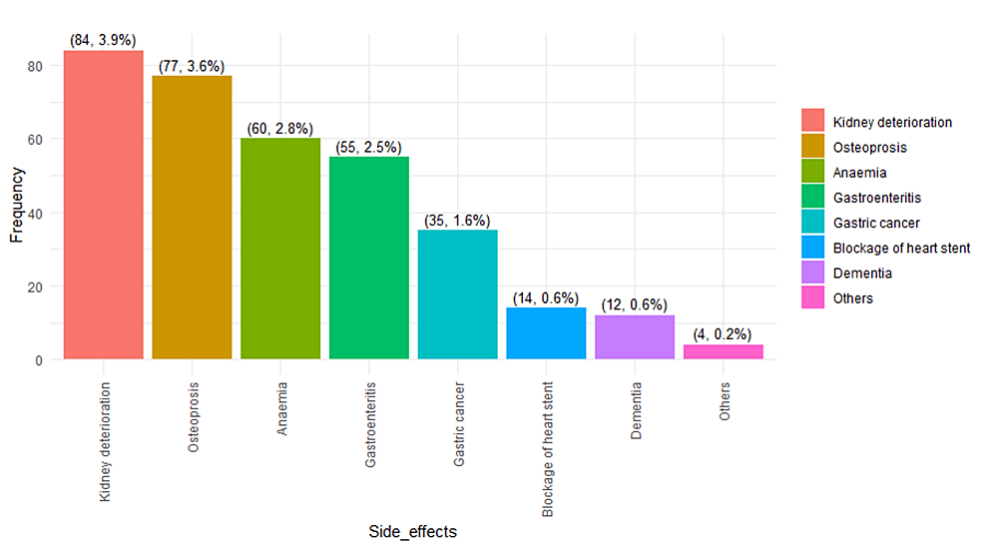
Bottom 8 side effects of PPI usage as identified in the study Notes: The data are presented in the form of frequencies (n) and percentages (%). PPI: proton pump inhibitor.

Figures 2-3 illustrate the top 8 and bottom 8 side effects identified in the study by participants. These figures represent a single illustration; however, due to space limitations, they are presented as two separate figures.

**Figure 4:**
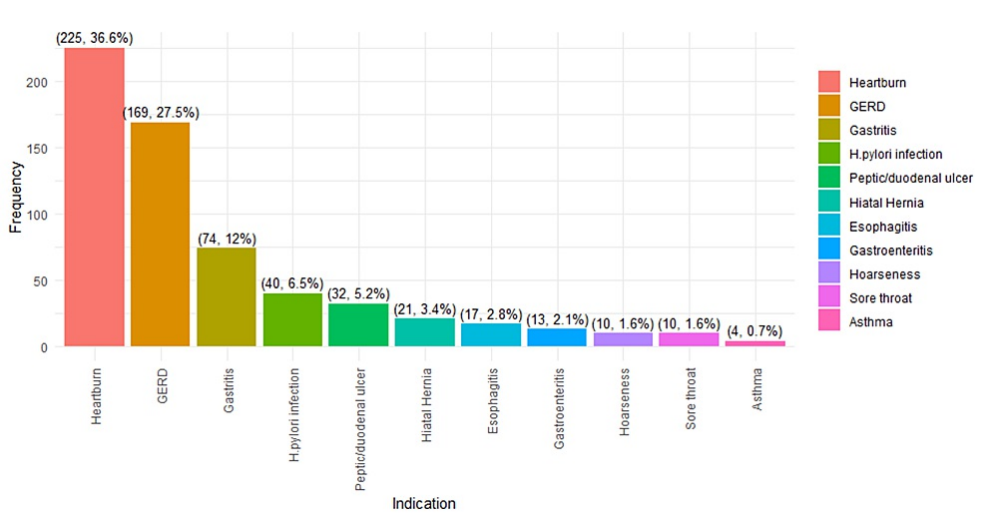
Comparison of identified indications for PPI usage Notes: The data are presented in the form of frequencies (n) and percentages (%). PPI: proton pump inhibitor.

Figure 4 illustrates the identified indications for PPI usage, with heartburn being the most common at 36.6%, while asthma is the least common at 0.7%.

Multivariate analysis

Multivariate analysis was conducted using the four independent variables in the study. The analysis was subsequently stratified by nationality, age, and education level, focusing on those with significant p-values in cross-tabulation.

Table 3 displays the probit analysis examining how the independent variables (side effects, widespread use, usage, and optimal timing) impact the dependent variable (awareness of antacids/PPIs), while Table 4 displays the probit analysis based on nationality.

**Table 3:** Probit analysis on awareness of antiacids/PPIs Notes: *p<0.1; **p<0.05; ***p<0.01; AdjOR: adjusted odds ratio; CI: confidence interval; PPI: proton pump inhibitor.

	Dependent variable: awareness of antiacids/PPIs		
Independent variable	AdjOR	CI	p-value
(1) PPIs cause side effects	1.188***	(1.076–1.313)	0.001
(2) PPIs are widely used	1.242***	(1.121–1.376)	0.00004
(3) Use of antiacids/PPIs	2.469***	(2.177–2.817)	<0.001
(4) Suitable time for PPIs	1.302***	(1.134–1.495)	0.0002
Constant	0.310	(0.233–0.410)	<0.001
Observations	1058		
Log likelihood	–529.6		
Akaike Inf. Crit.	1069		

**Table 4:** Probit analysis based on nationality Notes: *p<0.1; **p<0.05; ***p<0.01; AdjOR: adjusted odds ratio; CI: confidence interval; PPI: proton pump inhibitor.

	Dependent variable: Awareness of PPIs among Saudis		
Independent variable	AdjOR	CI	p-value
(1) PPIs cause side effects	1.183***	(1.070–1.308)	0.002
(2) PPIs are widely used	1.243***	(1.120–1.379)	0.00004
(3) Use of antiacids/PPIs	2.430***	(2.140–2.775)	<0.001
(4) Suitable time for PPIs	1.286***	(1.118–1.480)	0.005
Constant	0.318	(0.239–0.422)	<0.001
Observations	1023		
Log-likelihood	–519		
Akaike Inf. Crit.	1047		

Tables 5-7 showcase the stratified results categorized by working in the medical field, age, and education. The provided values include adjusted odds ratios, corresponding confidence intervals, and associated p-values. Results are interpreted within 90%, 95%, and 99% confidence intervals. It is essential to clarify that, for this study's context, the baseline confidence interval was set at a 95% confidence level and above.

**Table 5:** Probit analysis based on experience working in the medical field Notes: *p<0.1; **p<0.05; ***p<0.01; AdjOR: adjusted odds ratio; CI: confidence interval; PPI: proton pump inhibitor.

Independent variable	Dependent variable: awareness of antiacids/PPIs			
	Yes	p-value	No	p-value
(1) PPIs cause side effects	1.264*	0.053	1.105*	0.094
	(0.998–1.600)		(0.983–1.241)	
(2) PPIs are widely used	1.059	0.626	1.268***	0.0002
	(0.835–1.335)		(1.122–1.435)	
(3) Use of antiacids/PPIs	1.675***	0.002	2.895***	<0.001
	(1.244–2.357)		(2.514–3.355)	
(4) Suitable time for PPIs	1.537**	0.012	1.209**	0.022
	(1.105–2.157)		(1.025–1.425)	
Constant	0.698	0.29	0.263	<0.001
	(0.358–1.361)		(0.187–0.368)	
Observations	257		801	
Log-likelihood	–105		–362	
Akaike Inf. Crit.	220		733	

**Table 6:** Probit analysis based on age categories Notes: *p<0.1; **p<0.05; ***p<0.01; AdjOR: adjusted odds ratio; CI: confidence interval; PPI: proton pump inhibitor.

Independent variable	Dependent variable: awareness of PPIs among Qassim residents				
	<20 yrs (1)	21–30 yrs (2)	31–40 yrs (3)	41–50 yrs (4)	51–60 yrs (5)
(1) PPIs cause side effects	1.238	1.297***	1.011	1.16	1.17
	(0.929–1.655)	(1.108–1.519)	(0.766–1.332)	(0.929–1.446)	(0.787–1.755)
(2) PPIs are widely used	1.178	1.218**	1.172	1.455***	0.794
	(0.890–1.561)	(1.042–1.424)	(1.883–1.561)	(1.137–1.868)	(0.497–1.226)
(3) Use of antiacids/PPIs	1.919***	2.902***	6.716***	2.242***	3.527***
	(1.404–2.667)	(2.189–4.027)	(3.308–22.60)	(1.782–2.855)	(2.299–5.762)
(4) Suitable time for PPIs	1.279	1.565***	1.331	0.94	2.034**
	(0.887–1.843)	(1.256–1.955)	(0.923–1.921)	(0.683–1.285)	(1.037–4.138)
Constant	0.260***	0.249***	0.339***	0.405**	0.145***
	(0.125–0.525)	(0.157–0.390)	(0.169–0.667)	(0.199–0.806)	(0.032–0.545)
Observations	132	432	183	224	75
Log-likelihood	–72.174	–223.982	–70.83	–104.222	–25.761
Akaike Inf. Crit.	154.348	457.965	151.66	218.443	61.521

**Table 7:** Probit analysis based on the level of education Notes: *p<0.1; **p<0.05; ***p<0.01; AdjOR: adjusted odds ratio; CI: confidence interval.

Independent variable	Dependent variable: awareness of PPIs among Qassim residents				
	High school (2)	Diploma (3)	Bachelor (4)	Higher degree (5)
(1) PPIs cause side effects	1.013	1.106	0.986	1.239***	1.533
	(0.702–1.458)	(0.904–1.352)	(0.669–1.436)	(1.083–1.417)	(0.850–2.932)
(2) PPIs are widely used	0.996	1.245**	1.481**	1.257***	1.017
	(0.660–1.479)	(1.021–1.520)	(1.000–2.224)	(1.089–1.449)	(0.518–1.918)
(3) Use of antiacids/PPIs	3.643***	2.522***	3.386***	2.324***	2.547**
	(2.006–7.348)	(1.941–3.379)	(2.180–5.969)	(1.975–2.760)	(1.304–6.268)
(4) Suitable time for PPIs	0.827	1.256	1.35	1.326***	3.011**
	(0.480–1.355)	(0.948–1.665)	(0.757–2.382)	(1.097–1.602)	(1.311–9.277)
Constant	0.657	0.398***	0.200***	0.278***	0.136***
	(0.252–1.692)	(0.225–0.696)	(0.063–0.571)	(0.186–0.411)	(0.021–0.672)
Observations	72	260	96	587	43
Log-likelihood	−35.499	−132.398	−32.768	−302.722	−14.168
Akaike Inf. Crit.	80.997	274.795	75.536	615.444	38.337

## Discussion

Proton pump inhibitors are frequently prescribed medications for managing GERD and related conditions. Despite their established effectiveness, concerns have arisen regarding potential side effects and misuse. This study assesses the awareness and understanding of PPIs among the general population in Saudi Arabia's Qassim Region. The research examines PPI awareness, side effects, widespread usage, and optimal timing for antacid/PPI use.

In the realm of PPIs and their potential side effects, the current study unveils a striking connection. It delves into the relationship between participants' awareness of antacids and PPIs and their perspectives on the potential short-term side effects of PPIs. An adjusted odds ratio (adjOR) of 1.19 indicates that those believing in minimal side effects from short-term PPI use are about 1.19 times more familiar with these medications compared to others. The majority of participants (34.3%) who were aware of PPIs believed that short-term usage of PPIs would not lead to side effects. In total, 52.6% perceived that short-term PPI usage was unlikely to cause side effects, while 14.9% believed it could, leaving 32.4% uncertain, indicating limited recognition of PPIs' side effects. These findings parallel Aljahdil's [7] study, which found that most participants (43.6%) were unaware of the side effects. The present study documented that a majority of patients (22.8%) lacked awareness of any side effects linked to PPI usage, in contrast to those who pinpointed nausea and vomiting as the primary side effects of PPI usage (14.5%). These results concur with the observations made by Hamurtekin et al. [19] and White et al. [20] studies, both of whom highlighted that most patients were not unaware of potential adverse effects associated with PPIs. While participants' medical backgrounds did not yield statistically significant differences, the age category of 21-30 years showed notable results. Within this group, individuals were more likely to hold the belief that short-term PPI use does not lead to side effects, distinct from other age groups. Notably, this correlation was not strong in other age brackets due to potential variations in information exposure, health beliefs, or generational perspectives. Similarly, the education-level analysis highlighted a significant adjOR for the bachelor's degree category (1.24). This suggests that individuals with a bachelor's degree were likelier to know about antacids/PPIs when perceiving minimal side effects. This emphasizes education's role in understanding medication usage and side effects, with implications for healthcare communication and patient education.

Awareness of antacids/PPIs can influence perceptions of the extent of PPI usage. Familiarity with antacids/PPIs enhances understanding of their purposes, benefits, and risks. This awareness informs viewpoints on suitable PPI use scenarios, shaping perceptions of their prevalence. In this study, the results revealed a statistically significant correlation between participants' awareness of antacids/PPIs, as well as their perceptions of the extent of PPI usage. The adjOR of 1.24 indicated that individuals who believed in extensive PPI usage were around 1.24 times more likely to have familiarity with these medications, compared to those whose beliefs did not match this perspective. The ongoing study has unveiled a compelling connection between PPI awareness and their utilization within the Qassim region (45.1%), in contrast to those unaware of PPIs (20.6%). These figures find their roots in the accessibility of PPIs as over-the-counter medications, as noted in the study by Jaynes and Kumar [12]. Furthermore, these findings underscore the prominence of PPI usage, ranking among the top ten most frequently employed drugs in Saudi Arabia, a validation documented in the study by Alkhamees et al. [23]. The five primary reasons for the widespread usage of PPIs among the residents of Qassim were heartburn (36.6%), gastroesophageal reflux disease (27.5%), gastritis (12%), H. pylori infection (6.5%), and peptic ulcers (5.2%). Although the percentages were not closely aligned, a similar pattern in the reasons for PPI usage in Saudi Arabia was reported in the AlJahdli [7] study: heartburn (56.4%), gastroesophageal reflux disease (51.1%), gastritis (21.8%), H. pylori infection (20%), and peptic ulcers (15.7%).

Digging deeper, the data were split by participants' education levels: less than high school, high school, diploma, bachelor's, and higher degrees. Notably, adjOR for high school (1.25), diploma (1.48), and bachelor's (1.26) showed significance. Importantly, bachelor's degree holders showed a highly significant association compared to high school and diploma qualifications. These findings highlight the link between participants' education and awareness of antacids/PPIs in PPI usage perceptions. The strong significance among bachelor's degree holders underscores higher education's role in shaping understanding. These insights imply tailored healthcare communication strategies for diverse educational backgrounds. This finding is in agreement with the studies by Aljahdli [7] and Asdaq [24] in Saudi Arabia and the Haastrup et al. [29] study in Denmark, all of which demonstrated significant associations between awareness of PPIs, their prior usage, and educational attainment. The study reported that knowledge of PPIs was statistically significant among individuals with a bachelor's degree.

Regarding the perception of widespread antacid/PPI usage, only the adjOR for the age groups 21-30 years (1.22) and 41-50 years (1.46) showed statistical significance. This implies that individuals within these age brackets may hold different viewpoints about the prevalence of PPI usage in the Qassim region, significantly affecting their awareness of antacids/PPIs. This could stem from generational disparities in exposure to health information, evolving medical recommendations, or varying health concerns. Younger individuals (21-30 years) might be better informed due to greater access to online resources, while those in the 41-50 age group might be influenced by their life stage, leading to heightened awareness. These findings offer valuable insights into age-related variations in perceptions and awareness of PPI utilization. This discovery aligns with the results of the study by Aljahdli et al. [7], which also found a noteworthy correlation between older participants and a greater frequency of PPI knowledge in comparison to participants of younger age; the study by Van Boxel et al. [21] associated low educational attainment with chronic utilization of PPIs; the study by Alzahrani et al. [27] linked better knowledge on management of GERD to age; and the study by Freedberg et al. [18] associated the use of PPIs with a high risk of fracture among young adults in comparison to children.

When participants were categorized by their medical field background, the associations were as follows: among "yes" responders, the adjOR was 1.06, and the p-value was 0.626. Among "no" responders, the adjOR was 1.27, with a p-value of 0.0002. This indicates that participants with medical field experience had a higher adjOR for the "no" response, showing a clear link between their background and perceptions of PPI prevalence. Conversely, participants without medical experience had a lower adjOR for the "yes" response. These findings suggest participants' medical background could influence their views on PPI usage in Qassim, impacting their antacid/PPI awareness understanding. This finding resembles the results of Aljahidi's [7] study, which indicated that being employed in the medical field was linked to a greater level of awareness and usage knowledge regarding PPIs.

In the realm of participants' awareness of antacids/PPIs and their previous medication use, a significant and captivating connection comes to light. The adjOR of 2.47 illuminates this revelation, highlighting that those who have experienced these medications are approximately 2.47 times more likely to be acquainted with them compared to non-users. The exploration continues with a revelation of statistically significant prevalence rates of PPI utilization with medical consultation (p < 0.001) in the Qassim region. Among those previously aware of them, the prevalence stands at 24.5%. Conversely, the prevalence of usage without medical consultation is 9% (p = 0.006), while the prevalence among those initially unaware of PPIs reaches 24.3% (p = 0.05). These prevalence rates mirror those reported in Aljahdli's [7] study in Saudi Arabia. There, the prevalence of PPI usage with medical consultation was 25.7%, and usage without medical consultation was reported at 10.3%. The journey into the interconnected world of antacids/PPIs and their usage continues, shedding light on an essential facet of healthcare understanding. The usage findings correlate with the study by Basheikh et al. [22] in Saudi Arabia, which revealed a significant prescription rate for PPIs within an academic hospital. Similarly, the study by Alkhamees et al. [23] documented an increasing trend in PPI usage in Saudi Arabia. Moreover, the study by Asdaq et al. [24] conducted research in Saudi Arabia that highlighted doctors' heightened reliance on PPIs in comparison to pharmacists and fellow doctors. Additionally, the study by Alasmari et al. [25] explored the perspectives of physicians and pharmacists in Saudi Arabia, identifying a higher occurrence of inadequate knowledge juxtaposed with substantial knowledge concerning PPIs. Consequently, this current study signifies that doctors within Saudi Arabia's Qassim region contribute significantly to the elevated prescription of PPIs for their patients, similar to what has been documented in Luo et al. [4] and Savarino et al. [14,15] studies.

Comparing odds ratios based on participants' involvement in the medical field showed distinct patterns. "Yes" responders had an adjOR of 1.68, indicating a 1.68 times higher likelihood of being aware of antacids/PPIs among users, which is similar to the results reported in the study by Aljahdli et al. [7]. In contrast, "no" responders had a significantly higher adjOR of 2.90, suggesting a 2.90 times higher likelihood of awareness among non-users. Non-users showed a stronger link between lack of medication use and awareness, possibly due to active information seeking. Users might have gained awareness through personal experiences. This dynamic underscores the role of personal experience and professional background in shaping medication awareness. All odds ratios regarding antacid/PPI use were statistically significant across age groups. The ascending trend in adjusted odds ratios indicated increasing awareness with age. The significant odds ratios underscored the relationship between age and participants' awareness of these medications, implying age-influenced familiarity. The adjOR suggested both awareness and usage increased with age, which is similar to what was reported in the study by Aljahdli et al. [7] and Alzahrani et al. [27]. The rising trend in adjOR values across age groups indicated a higher likelihood of awareness and use as age advanced. Statistically significant odds ratios further supported the link between age, awareness, and usage. To delve deeper, the data were segmented by participants' educational levels: less than high school, high school, diploma, bachelor, and higher degree. Remarkably, across all educational categories, the adjOR showed statistical significance. However, those with lower education levels (less than high school, high school, and diploma) exhibited a larger adjOR than those with bachelor's and higher degrees. This implies a more pronounced use of antacids/PPIs among individuals with lower education levels, which aligns with what was reported in Van Boxel’s [21] study. These findings underline the correlation between educational background and awareness of antacids/PPIs in relation to PPI usage perceptions. The heightened significance among individuals with lower education levels highlights education's potential influence on medication usage perception. These insights suggest tailored healthcare communication for diverse educational backgrounds.

The timing for taking PPIs varies with the medication and individual condition. Generally, PPIs are taken before meals. They reduce stomach acid production, and taking them before eating allows for absorption and early action before food intake. The present study revealed a statistically significant relationship between participants' awareness of antacids/PPIs and their subjective opinions regarding the optimal time for medication intake. The calculated adjOR of 1.30 indicated that individuals expressing opinions about suitable medication timing were approximately 1.30 times more likely to be familiar with these medications compared to those not sharing such opinions. Among those aware of PPIs, 37.1% recognized the need to take PPIs before meals, 17.3% indicated after meals, and 3.6% mentioned with meals. When the data were not segregated, PPI usage before meals was recorded at 50.9%, after meals at 41.4%, and with meals at 7.7%, figures nearly in line with what was reported in the study by Aljahdil et al. [7: before meals (59.8%), after meals (39.3%), and with meals (0.9%). Studies such as Chen and Brandy [9] advocated the use of PPIs before or after meals, while Laine et al. [11] suggested their efficacy was heightened shortly before meals.

Categorized by participants' medical backgrounds, results showed: "yes" responses had an adjOR of 1.54, p-value = 0.012; "no" responses had an adjOR of 1.21, p-value = 0.022. This suggests a medical background might influence optimal medication timing perceptions and antacid/PPI awareness. Professionals prioritize timing due to expertise; non-medical individuals link awareness to personal health. These findings underscore medical knowledge's role in viewpoints and relevance for healthcare communication. The adjOR for age groups 21-30 years (1.565) and 51-60 years (2.034) on antacids/PPIs timing was statistically significant. These age ranges held distinct viewpoints, impacting awareness. Notably, those aged 21-30 and 51-60 had a stronger correlation between timing opinions and antacid/PPI awareness. This enhances understanding of age-related variations in perceptions and awareness. Regarding education and optimal timing perception linked to antacid/PPI awareness, only higher education levels showed significance. Higher degrees had a larger adjOR than bachelor's. This highlights stronger awareness among advanced education levels. These findings emphasize education's link to antacid/PPI awareness in medication timing perceptions. Higher degrees' significance underscores advanced education's influence. Insights suggest tailored communication strategies for diverse education levels.

Recommendations

The current study highlighted that a significant portion of individuals in the Qassim region lack awareness regarding the potential side effects of antacids/PPI usage. Healthcare professionals are recommended to acknowledge and explore patients' beliefs concerning the perceived absence of short-term PPI side effects. Engaging in discussions about patients' perspectives on side effects presents an effective approach to enhancing their awareness and comprehension of antacids and PPIs.

The strong association between PPI usage and its prevalence in the Qassim region might be influenced by individual perceptions and the accessibility of over-the-counter PPIs. Based on these findings, healthcare practitioners are encouraged to recognize the potential impact of patients' perceptions regarding PPI prevalence in the region. Integrating conversations about patients' viewpoints on medication prevalence can serve as a means to elevate their awareness and understanding of antacids/PPIs.

Medical practitioners were identified as the primary prescribers of PPIs, despite some individuals using PPIs without medical consultation. Considering these findings, healthcare practitioners should prioritize acknowledging the potential influence of prior medication usage when designing patient education and communication strategies. Incorporating individuals' personal experiences with medication usage can enhance the effectiveness of interventions aimed at raising awareness about antacids/PPIs.

Half of the study participants were aware of the optimal timing for PPI intake before meals, while the other half were either uncertain or identified during meals. Given these results, healthcare practitioners are advised to recognize the importance of patients' opinions on suitable medication timing. Incorporating discussions about appropriate timing in patient communication can significantly contribute to augmenting awareness and understanding of antacids/PPIs among Qassim region residents.

## Conclusions

In conclusion, this study offers significant insights into healthcare awareness within the Qassim region. The findings underscore a notable lack of awareness among a significant portion of the population regarding the potential side effects associated with antacid/PPI usage. The study reveals a strong correlation between PPI usage and its prevalence in the Qassim region. This link may be influenced by individual perceptions and the easy accessibility of over-the-counter PPIs. Understanding this relationship is crucial for designing targeted educational efforts to dispel misconceptions and improve overall awareness. Notably, medical practitioners emerge as primary prescribers of PPIs, alongside instances of non-medical PPI use. This highlights the need to acknowledge the pivotal role of healthcare professionals and to promote safe and informed medication practices among the public. Regarding the optimal timing for PPI intake, a divergence of opinions exists among participants. While half of the participants are aware of the preferred timing before meals, the remaining participants exhibit uncertainty or identify other timings. This variation underscores the potential for tailored communication strategies to enhance clarity around medication intake practices. Moreover, the study demonstrates that factors such as age, educational attainment, and employment within the medical field significantly influence awareness gaps concerning potential side effects, PPI prevalence, adherence to medical prescriptions, and optimal timing for PPI uptake. These demographic insights can inform the design of targeted interventions aimed at addressing specific knowledge gaps.

In summary, the findings emphasize the need for comprehensive healthcare education efforts to bridge awareness gaps and promote informed medication practices. By understanding the nuances of individual perceptions, medical involvement, and demographic influences, healthcare providers and policymakers can collaboratively work towards empowering individuals in the Qassim region to make informed decisions about their health and medication use.
